# Long-Range Genomic Enrichment, Sequencing, and Assembly to Determine Unknown Sequences Flanking a Known microRNA

**DOI:** 10.1371/journal.pone.0083721

**Published:** 2013-12-20

**Authors:** Zhaorong Ma, Michael J. Axtell

**Affiliations:** Integrative Biosciences PhD Program in Bioinformatics and Genomics, Huck Institutes of the Life Sciences, and Department of Biology, Pennsylvania State University, University Park, Pennsylvania, United States of America; East Carolina University, United States of America

## Abstract

Conserved plant microRNAs (miRNAs) modulate important biological processes but little is known about conserved cis-regulatory elements (CREs) surrounding *MIRNA* genes. We developed a solution-based targeted genomic enrichment methodology to capture, enrich, and sequence flanking genomic regions surrounding conserved *MIRNA* genes with a locked-nucleic acid (LNA)-modified, biotinylated probe complementary to the mature miRNA sequence. Genomic DNA bound by the probe is captured by streptavidin-coated magnetic beads, amplified, sequenced and assembled *de novo* to obtain genomic DNA sequences flanking *MIRNA* locus of interest. We demonstrate the sensitivity and specificity of this enrichment methodology in *Arabidopsis thaliana* to enrich targeted regions spanning 10–20 kb surrounding known *MIR166* and *MIR165* loci. Assembly of the sequencing reads successfully recovered all targeted loci. While further optimization for larger, more complex genomes is needed, this method may enable determination of flanking genomic DNA sequence surrounding a known core (like a conserved mature miRNA) from multiple species that currently don't have a full genome assembly available.

## Introduction

microRNAs (miRNAs) originate from primary transcripts called pri-miRNAs that are transcribed by RNA polymerase II. In plants, pri-miRNAs are processed into 20–24 nt mature miRNAs by the Dicer-like 1 (DCL1) protein, and then incorporated into RNA-induced silencing complexes (RISCs) which serve to negatively regulate target mRNAs [1]. Conserved plant miRNAs modulate important biological processes including development, immune responses, nutrient homeostasis and hormone responses [1]–[3]. The spatial and temporal control of miRNA accumulation needs to be fine tuned in order for plants to respond to ever-changing environmental and intracellular signals. This fine-tuning can be done either at the transcriptional level of *MIRNA* genes or the post-transcriptional level. In animals, post-transcriptional regulation of miRNA expression functions either via signaling pathways centered on the Microprocessor (the protein complex processing pri-miRNAs) or interaction between RNA-binding proteins and *cis*-regulatory sequences on the terminal loop of miRNA precursors [4]. In plants, it is known that core promoters exist and motifs related to development, stress responses, and hormonal control are over-represented at several loci [5], [6]; however, a full understanding of conserved *cis*-regulatory elements (CREs) surrounding plant *MIRNA*s requires additional studies.

Control of gene expression is partly conveyed by specific DNA sequences that act as CREs by recruiting transcription factors (TFs) or repressors [7], [8]. Conserved CREs have been discovered by sequencing multiple species followed by comparative genomics [9]–[13]. However, even with the advances in next generation sequencing technologies, sequencing and assembling multiple plant genomes is still beyond the resources of a typical lab. If the flanking genomic sequences of interest can be captured specifically in multiple species, identification of CREs need not require complete genome assemblies. To select and enrich the flanking genomic sequences surrounding *MIRNA* genes, we could exploit the fact that conserved *MIRNA*s always have nearly identical sequences in the 20–24 nt mature miRNA region in multiple plant species [2], [3]. A methodology which captures long, unknown genomic DNA sequences flanking a short known core sequence, the mature miRNA in this case, could be used to efficiently isolate the flanking DNA of interest from species that lack a reference genome assembly.

The idea of enriching and sequencing specific genomic regions of interest has been widely implemented. Strategies for targeted genomic enrichment include polymerase chain reaction (PCR) [14], molecular inversion probes (MIPs) ([15], [16]) and microarray capture ([17]–[21]). However, PCR requires the knowledge of two primer sequences flanking the region of interest, thus it is impossible to obtain unknown sequences flanking a single known core sequence. PCR also tends to lack robustness for sequences longer than ten kb [22]. Inverse PCR, a variant of PCR, can amplify unknown sequences flanking a known core sequence. It uses two primers oriented away from the core sequence to amplify the ligated flanking sequence following restriction digestion [23], [24]. The core sequence has to have a minimum length to allow the annealing of two non-overlapping primers. Thus, inverse PCR is unsuitable for amplifying flanking sequences of a mature miRNA region which is 20–24 nt in length. Target capture with MIPs uses a single-stranded oligonucleotide consisting of a common linker flanked by target-specific sequences to anneal to the target DNA, followed by “gap-filling” between the target-specific sequences with a DNA polymerase, and finally amplifies by PCR with primers directed at the common linker [15], [16]. MIPs also require two known sequences, and the capture uniformity is relatively poor [22], [25]. Microarray hybrid capture, using probes against sequences of interest ([17]–[21]), is inefficient for capturing extremely long sequences flanking a short known sequence [19]. To overcome many of the above shortcomings, solution-based target enrichment methods have been developed, which apply similar principles as microarray-based capture using specific probes designed to the targeted regions of interest. Solution-based target enrichment uses an excess of probes over genomic DNA, which drives the hybridization further to completion with a smaller amount of genomic DNA than microarray-based capture [22]. Also, solution-based capture can be performed in micro-centrifuge tubes or 96-well plates, which is easily scalable compared to microarray capture. To date, the major application of solution-based capture is exon targeting followed by SNP finding [26], [27]. However, the current application of solution-based capture uses long RNA probes of several hundred bases in length to cover the full lengths of exons, and the design of the probes requires a fully sequenced reference genome, or at least the exon sequences of interest.

We developed a novel solution-based targeted enrichment methodology to rapidly capture, enrich and sequence a large, unknown genomic region flanking a small known target of interest. In this study, we tested the strategy with a 21 nt probe against the miR166 mature sequence in *Arabidopsis thaliana*, and found that this methodology was highly specific and sensitive to enrich regions flanking the targeted loci. *de novo* assembly of the reads sequenced from the enriched sample successfully assembled all targeted loci into long contigs. We propose that the further development of this method may enable us to easily obtain flanking genomic DNA surrounding short conserved regions (like mature miRNAs) in multiple plant taxa that lack complete genome assemblies, and in turn accelerate discovery of CREs surrounding such loci.

## Results

### Enrichment of an ∼20 kb region flanking *Arabidopsis MIR166a*

**Figure 1:**
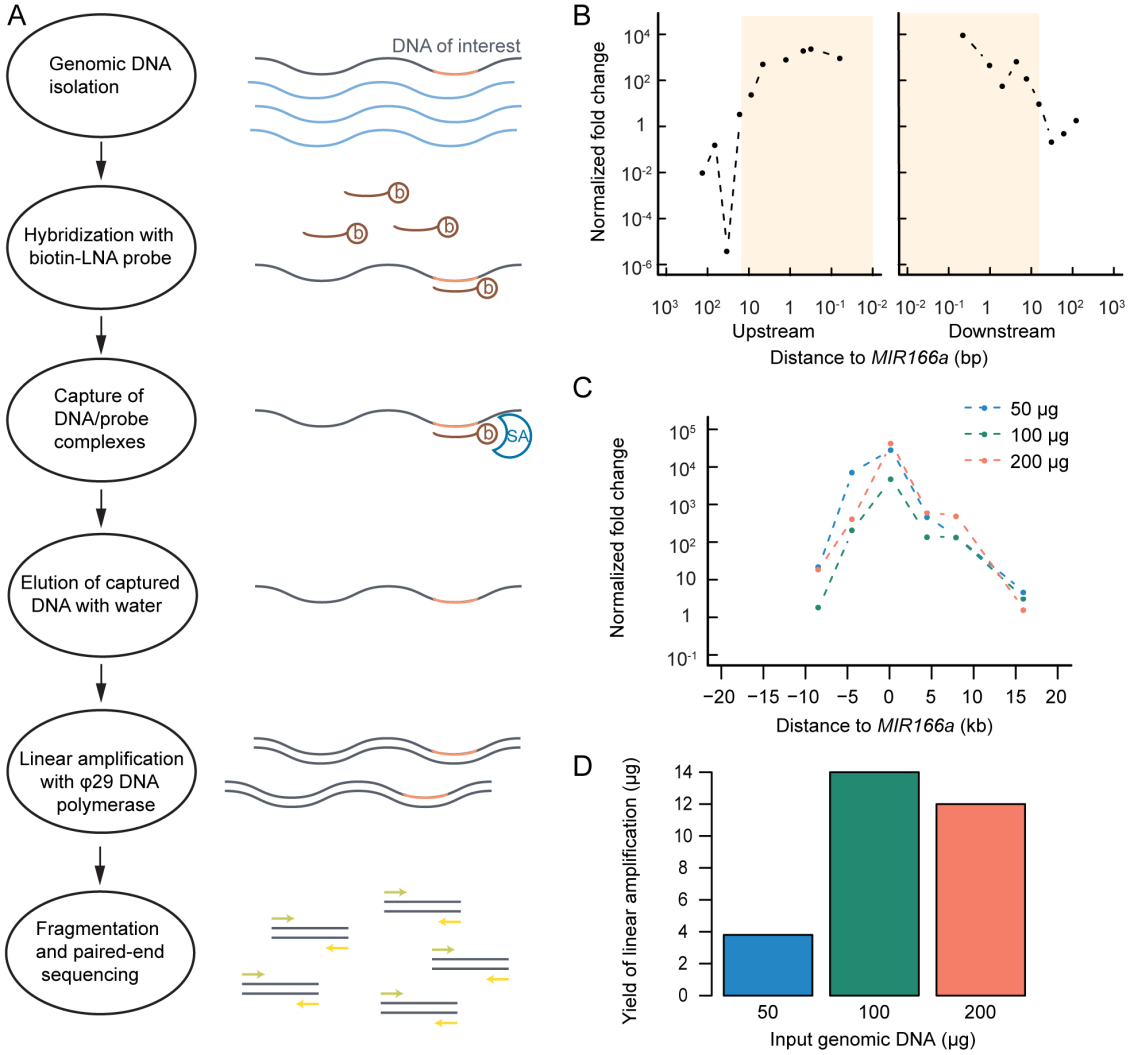
Pilot targeted enrichment experiment in *Arabidopsis* shows enrichment near a targeted locus. (A) Schematic overview of targeted genome enrichment method. b: Biotin, SA: Streptavidin. (B) Quantitative real-time PCR (qPCR) of enriched DNA with designed primers surrounding the *MIR166a* locus. Normalized fold change relative to *Act1* (as a control) after enrichment is shown. Shaded box indicates the region with a normalized fold change above one. (C) Amount of input genomic DNA (gDNA) does not affect the fold of the enrichment. Normalized fold change relative to *Act1* after enrichment is shown with varying amount of gDNA. (D) Amount of gDNA affects the yield of the enrichment. Yield after enrichment is shown, as is measured by Qubit® Fluorometer.

The enrichment methodology is outlined as follows (Fig. 1A): Genomic DNA is hybridized with a biotinylated locked nucleic acid (LNA)-modified capture probe. Targeted genomic fragments paired with the probe are retained by binding to paramagnetic, streptavidin coated-beads while unbound fragments are washed away. Then the targeted fragments are eluted in hot water, subject to linear amplification by the DNA polymerase Φ29 and subsequently fragmented, sequenced and assembled.

A pilot enrichment experiment was performed with *Arabidopsis* genomic DNA and a 21 nt, biotinylated LNA capture probe complementary to the mature miR166 DNA sequence. The relative fold-enrichment of the targeted loci compared to a control region was determined with quantitative real-time PCR (qPCR) performed on the enriched and Φ29-amplified DNA. The pilot experiment successfully yielded enrichment in a region of ∼20 kb flanking the *MIR166a* locus, with a peak enrichment above 1,000-fold (Fig. 1B).

To optimize the enrichment protocol to increase final DNA yield, input genomic DNA concentrations, washing conditions, and linear amplification times were varied, and relative fold of enrichment at *MIR166a* was determined by qPCR. The optimized protocol is described in Methods. We found that changing the φ29 amplification time to two hours or more increased the final quantity of DNA (data not shown) without affecting enrichment (Fig. S1). Increasing the input amount of genomic DNA in the hybridization step of the targeted genomic enrichment by two-fold increased the final yield of enriched DNA product by four-fold (Fig. 1D) without lowering enrichment (Fig. 1C), while using an even larger amount of the input DNA did not further increase the total DNA yield (Fig. 1D). Overall, 100 µg genomic DNA input in the targeted enrichment followed by a two-hour φ29 amplification resulted in over 10 µg enriched DNA, enough for a high-throughput sequencing run which typically requires approximately one μg DNA.

### Successful enrichment at all *MIR166* and *MIR165* loci

An *Arabidopsis* genomic DNA sample prepared with the optimized targeted enrichment protocol was fragmented to an approximate mean size of 400 bp and sequenced on one lane of an Illumina GAIIx sequencer. The goal of sequencing the enriched sample was two fold: first, the sequencing reads were mapped back to the reference genome to evaluate the performance of the targeted enrichment methodology; second, the reads were *de novo* assembled with the Velvet assembly software [28] and parameters of the assembler tuned to optimize assembly quality. We obtained ∼25 million pairs of 76 nt paired-end reads, of which ∼18 million were mapped to the *Arabidopsis* genome (Table S1). 65.1% of the mapped reads mapped to the nuclear genome, 32.4% to the plastid genome and 2.5% to the mitochondrial genome.

**Figure 2:**
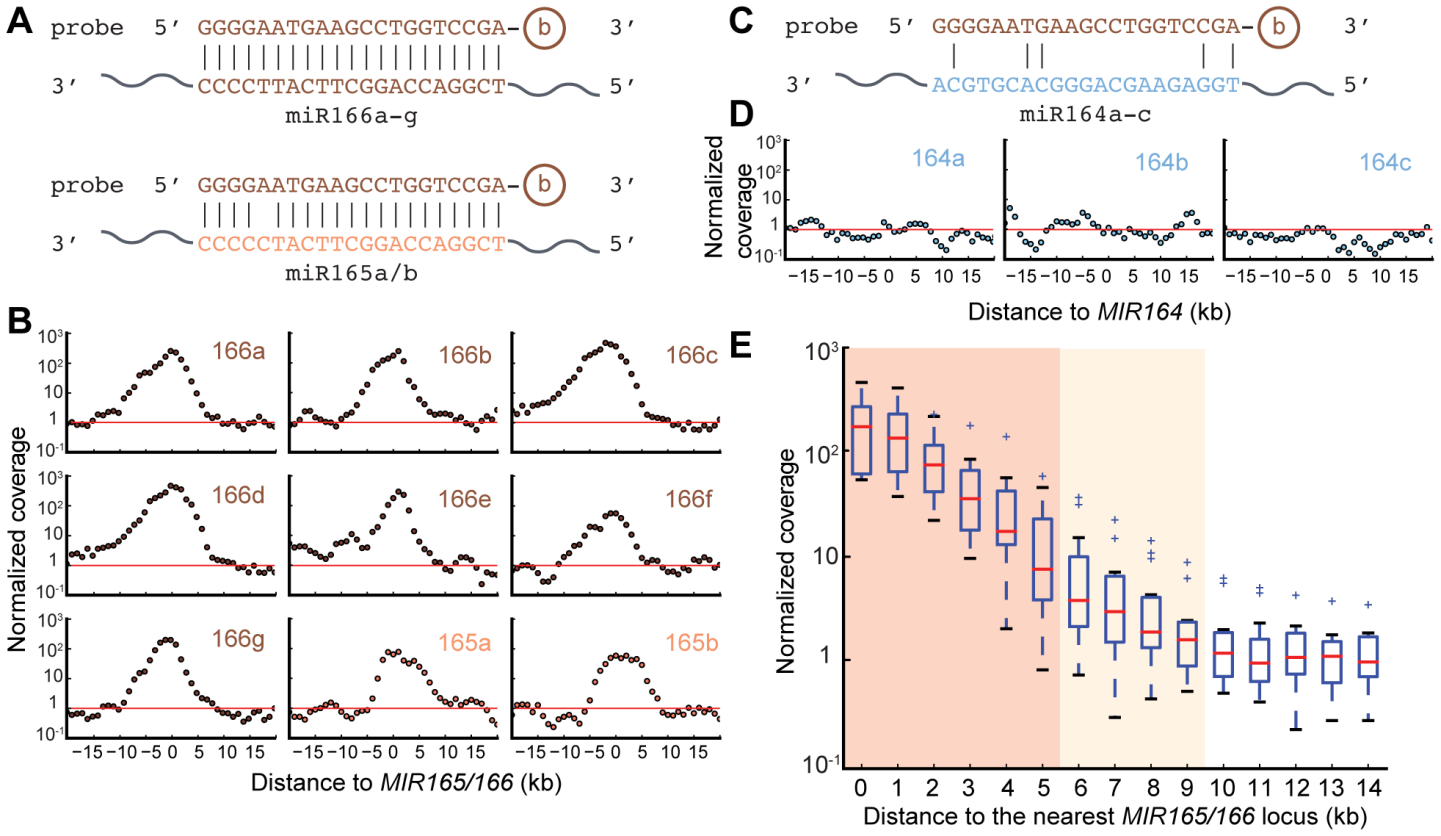
Enrichment in a 10 kb region flanking the targeted *MIRNA* loci. (A) Sequence alignments between capture probe and miR166/miR165 respectively. b: Biotin. (B) Normalized coverage at each 1 kb-sized bin flanking the indicated *MIRNA* loci. Red horizontal line indicates the genome average of the normalized coverage, which equals one. (C) As in (A) for miR164, which is not targeted by the probe. (D) As in (B) for *MIR164* loci, which are not targeted by the probe. (E) Regions of +/− 9 kb flanking the target sites are enriched. Box plot shows fold of enrichment of bins with increasing distance to the target sites. This is a tallied view of the nine individual targeted loci shown in (B). “+” symbols represent outliers that are outside 1.5 IQR (inner quartile range). Dark shade denotes p<0.01 with Student's t test against a normalized coverage of one. Light shade denotes p<0.05.

Mapped reads were tallied into 1 kb-sized bins and read coverage of each bin was calculated. The average read coverage per bin for the nuclear genome was 98 reads, compared to 36,433 for the plastid genome and 748 for the mitochondrial genome. The deep coverage of the organellar genomes is expected based on their high copy numbers relative to the nuclear genome and their small sizes. To achieve the first goal of evaluating the enrichment methodology, bins from organellar genomes were discarded, keeping only bins in the nuclear genome. Coverage of each bin was normalized to the nuclear genome average (termed normalized coverage). Enrichment is implied when the normalized coverage is above one. There are seven *MIR166* loci with perfect matches to the probe, and two *MIR165* loci with a single mismatch to the probe (miR165 and miR166 are highly similar miRNA families; Fig. 2A). Enrichment was observed in an approximately ten kb region flanking all targeted loci (Fig. 2B). A peak enrichment of 100-fold or more was evident for the seven *MIR166* loci in the genome with full complementarity to the capture probe, while a slightly lower peak of enrichment was evident for both *MIR165* loci in the genome which have one mismatch to the probe (Fig. 2A–B). As a control, three *MIR164* loci which have no significant complementarity to the probe were analyzed and indeed showed no evidence of enrichment (Fig. 2C–D).

In order to estimate the size of the enriched regions, Student's *t*-tests were performed to test the hypothesis that the mean normalized coverage of bins with increasing distances from one target site is not different from one. Normalized coverages of bins that were within nine kb from any one of the target sites were different from one with statistical significance (p<0.05), indicating that the size of the enriched regions was about 19 kb on average (totaling 135 kb for the eight targeted loci, *MIR166c* and *MIR166d* considered as a single locus as they are just two bins apart). Bins that were within five kb of the targets had an mean normalized coverage different from one with p<0.01, corresponding to a size of 11 kb significantly enriched regions (totaling 79 bins for eight targeted loci; Fig. 2E).

### Enrichment is both sensitive and specific

**Figure 3:**
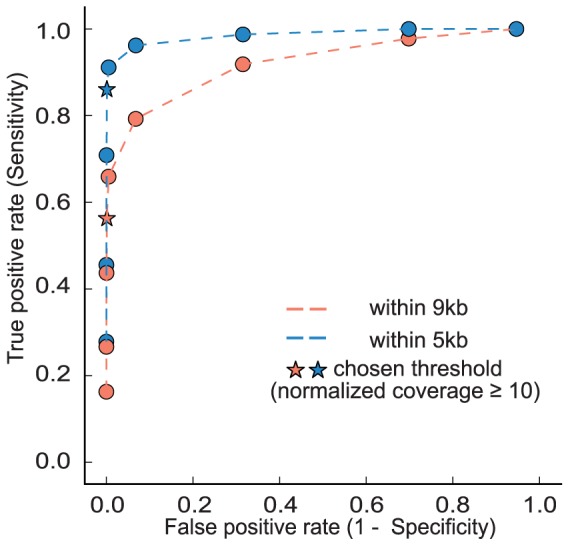
Performance analysis to determine enriched regions. Receiver operating characteristic (ROC) curves are shown with varying thresholds of normalized fold change, using within-9 kb or within-5 kb bins from target sites, respectively, as positives. Values shown in Table S2.

Next, the enrichment pattern was assessed across the genome, focusing on all “enriched” regions regardless of whether or not they were *MIR166* or *MIR165* loci. In order to determine the threshold of normalized coverage above which a bin could be defined as “enriched”, the sensitivity and specificity of the enriched bins at different thresholds were evaluated. The 79 bins within five kb away from any target sites were defined as positives. All other bins (totaling 119,070 bins) in the nuclear genome were considered negatives. Thus, a true positive was defined as a bin above the threshold of normalized coverage and within five kb from any target sites, while a false positive was defined as a bin above the threshold but outside the +/− five kb window. A true negative was defined as a bin below the threshold and outside of the +/− five kb region, while a false negative was defined as a bin within the +/− five kb region but below the threshold. By decreasing the threshold of normalized coverage of each bin, sensitivity increased while specificity decreased as expected (Table S2, Fig. 3). The same analysis was performed with the 135 within-9 kb bins as true positives (Table S2, Fig. 3). Sensitivity of the latter was not as high as the former at each threshold of normalized coverage, which is partly because the set of within-9 kb bins is less stringent (although the mean enrichment of all these bins is statistically significant, many bins in this set are in fact not enriched). We chose a normalized coverage of ten as the threshold of enrichment for further analysis, which maintained both sensitivity and specificity at relatively high levels. It is worth noting that at the chosen threshold, the false discovery rate is quite high (153/221), however, false positives (i.e. enriched regions not close to the targeted loci) are not a major concern for downstream analysis, because false positives, when later assembled into contigs during *de novo* assembly, will lack the sequence targeted by the probe (i.e. mature miR165/166).

### Targeted regions can be discriminated from sporadically enriched loci

**Figure 4:**
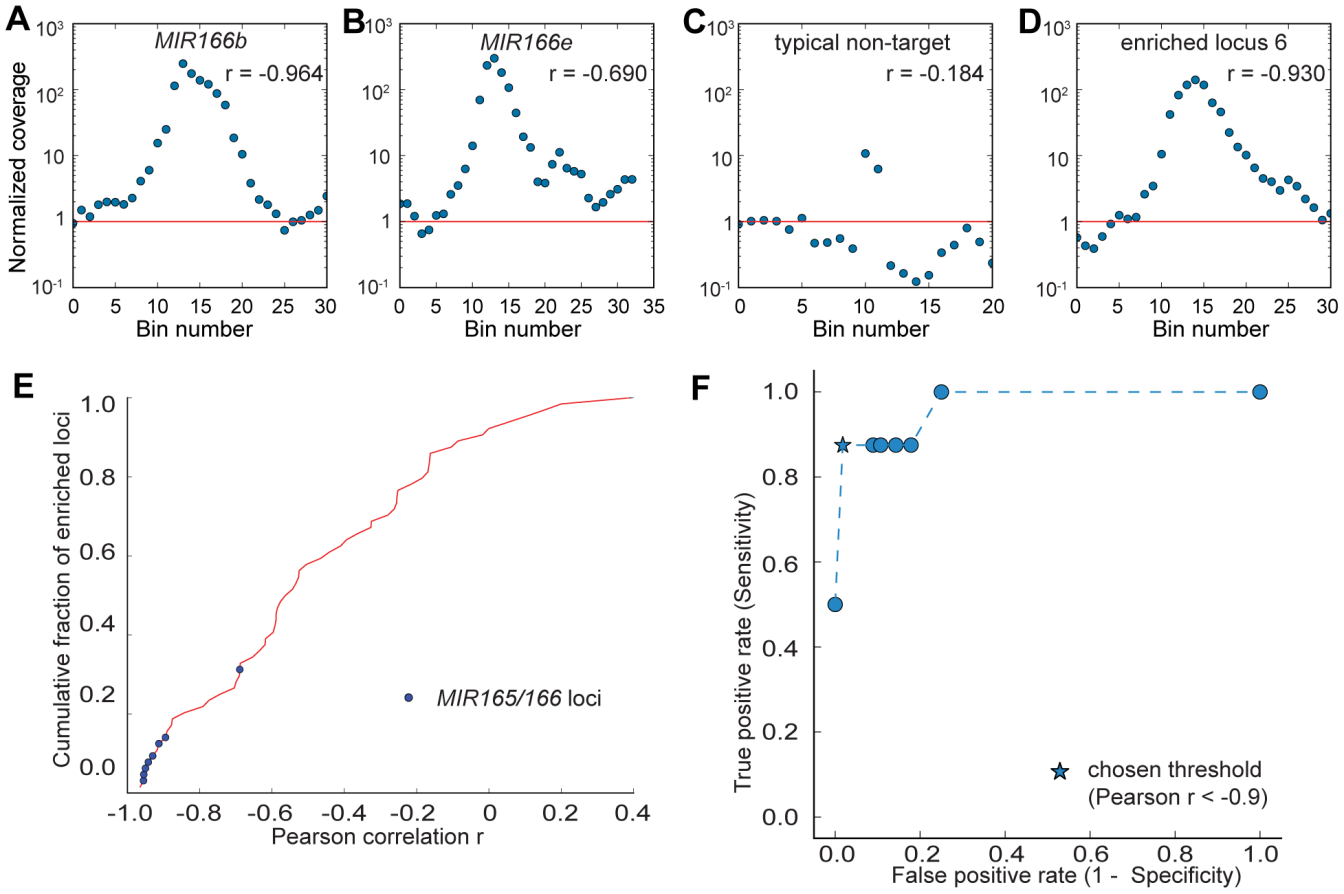
Targeted regions have a distinctive enrichment pattern. (A–D) Each panel shows the normalized coverage at each 1 kb-sized bin centered on a highly enriched bin. Pearson correlation r of |x| and log(y) is shown, where x is the distance to the most highly enriched bin in the region and y is the normalized coverage. Red line indicates the genome average of the normalized coverage, which equals one. See Fig. S2 for full details. (A) Region surrounding *MIR166b* targeted locus. (B) Region surrounding *MIR166e* targeted locus. (C) A typical region surrounding a non-targeted locus. (D) Region surrounding enriched locus 6, which is not a *MIR166* nor a *MIR165* locus. (E) Cumulative distribution of the Pearson correlation r for all 64 highly enriched regions. Blue dots indicate targeted *MIR165/166* loci. (F) Performance analysis to determine the optimized threshold of r to classify targeted and non-targeted regions. ROC curve is shown with varying threshold of r. Star-shaped dot indicates the chosen threshold of r = −0.9.

In order to examine the pattern of enriched genomic regions, bins with a normalized coverage above ten were merged if they were within ten kb apart, and extended ten kb on each side to examine the genomic landscape surrounding the enriched regions. After merging and extending, a total of 64 highly enriched regions were generated (Fig. S2), including all eight *MIR165/166* loci (*MIR166c* and *MIR166d* are closely linked on chromosome five, and as such were merged into a single locus in this analysis). When observing the landscape of adjacent bins centered on a highly enriched bin, *MIR165/166* flanking regions all exhibited a bell shape, reflecting lower enrichment further away from the probe binding site (Fig. 4A–B, Fig. S2, shaded panels), while other enriched regions generally showed only one or two highly enriched bins flanked by regions with a coverage close to the background level, possibly due to random amplification during sequencing or unannotated copy number variation relative to the reference genome assembly (Fig. 4C, Fig. S2, unshaded panels). In order to distinguish targeted regions from non-targeted regions based on the enrichment pattern in the surrounding regions of highly enriched bins, the Pearson correlation coefficient r was calculated to examine the linear dependence between |x| and log(y) where x is the distance to the most highly enriched bin in the 21 kb region centered on that bin and y is the normalized coverage (Fig. 4E, Table S3). The hypothesis is that if the region is centered on a real target site, enrichment should decrease exponentially as it moves further away from the target site. On the other hand, if the region is not targeted, no such correlation should be observed. To test this hypothesis, sensitivity and specificity was assessed with varying thresholds of r as the classifier of targeted and non-targeted regions (Fig. 4F). As expected, sensitivity increases while specificity decreases as the threshold of r increases (i.e. becomes less negative, indicating a weaker linear relationship). We chose r<−0.9 as the threshold to distinguish non-targeted from targeted regions. With this threshold, seven out of the eight enriched regions flanking *MIR165/166* loci were recovered (Fig. 4E–F, Fig. S2), the only exception being the *MIR166e* locus (Fig. 4B), possibly due to the secondary non-specific peak near the targeted locus confounding the linear dependence pattern. All other regions had r>−0.90 (a typical example is shown in Fig. 4C) except one: enriched locus 6 with genome coordinates chr1: 10314k–10344k (Fig. 4D, Table S3). Overall, a Pearson correlation test with threshold of r<−0.90 resulted in a sensitivity of 7/8 and specificity of 55/56, which is a sensitive and specific classifier of targeted and non-targeted loci. The above analysis demonstrates that the targeted enrichment methodology is highly specific to enrich a relatively long region flanking the targeted loci.

### Enrichment requires a high amount of probe complementarity

**Figure 5:**
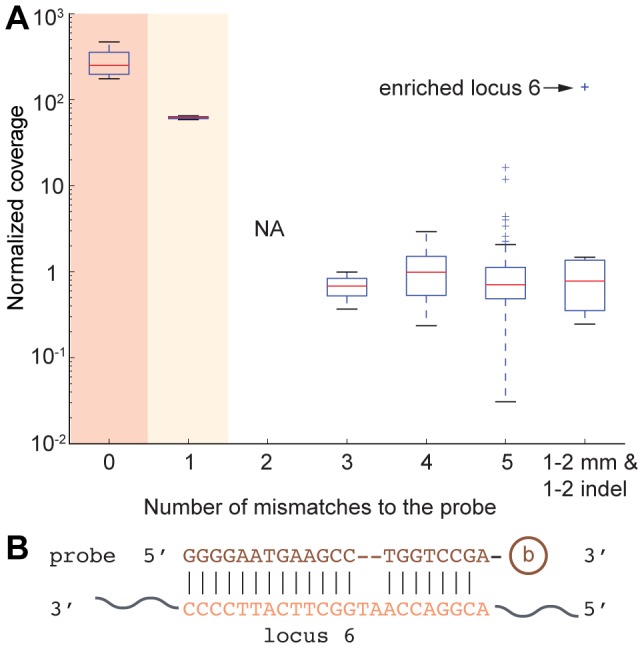
Enrichment is highly specific for loci with zero or one mismatch. (A) Box plot shows normalized coverage of loci with different mismatches to the probe. Last box shows genomic loci which are similar to locus 6, with one or two mismatches and one or two insertions and deletions in the alignment to the probe. “+” symbols represent outliers that are outside 1.5 IQR (inner quartile range). Dark shade denotes p<0.01 with Student's t test against a normalized coverage of one. Light shade denotes p<0.05. (B) Sequence alignment between capture probe and enriched locus 6.

We next analyzed how mismatches between potential targets and the probe affected enrichment. As slight sequence variation exists even for deeply conserved plant miRNAs, it is important to know how much sequence variation in the targeted sites can be tolerated. Therefore, normalized coverage at genomic loci with zero to five mismatches to the capture probe was examined, disallowing insertions or deletions (indels). All the loci with zero or one mismatches are *MIR165* or *MIR166* loci, and Student's t test revealed that the mean normalized coverage of loci with perfect complementarity and with one mismatch were both significantly different from the null hypothesis of one with p-values <0.01 and <0.05, respectively (Fig. 5A). No locus in the genome had exactly two mismatches to our probe. Genomic loci with three, four or five mismatches to the probe showed no enrichment, as the normalized coverage was not statistically different from the genome average. None of the 56 false-positive enriched loci (Fig. S2) had potential probe complementarity sites with between zero and four mismatches, and only one had sites with five mismatches, emphasizing that the reasons for sporadically enriched loci are likely not due to probe hybridization. This demonstrates that our strategy is generally specific to loci with zero, one, and perhaps two mismatches to the probe.

We next examined in closer detail enriched locus 6, which was the sole enriched locus that showed a robust bell curve of enrichment despite not being a *MIR166* or *MIR165* locus (Fig. 4D). Enriched locus 6 resides in the intergenic region between *AT1G29540.1* (unknown protein) and *AT1G29550.1* (eukaryotic initiation factor 4E protein). This enriched locus had no sequence similarity to the *MIR165/166* flanking regions (+/− 5 kb), nor did it exhibit similarity to rRNA sequences, thus ruling out simple explanations for its enrichment. We did identify a rather poor complementary site with a 5′ A-A mismatch, and a central two nt bulge (Fig. 5B). However, this is unlikely to be responsible for the enrichment of locus 6: Out of the six genomic loci which had one or two mismatches and one or two indels to the probe, enriched locus 6 was the only one with significant enrichment (Fig. 5A). Our *de novo* sequencing confirmed the sequence at this site was identical to the reference genome, ruling out the possibility of an un-annotated indel that created a perfect probe complementarity site. We currently do not understand the reason why this locus was enriched. However, it is the single exception to the general rule that robust enrichment requires high complementarity to the probe.

### *de novo* assembly accurately recovers genomic sequences flanking targeted loci

**Figure 6:**
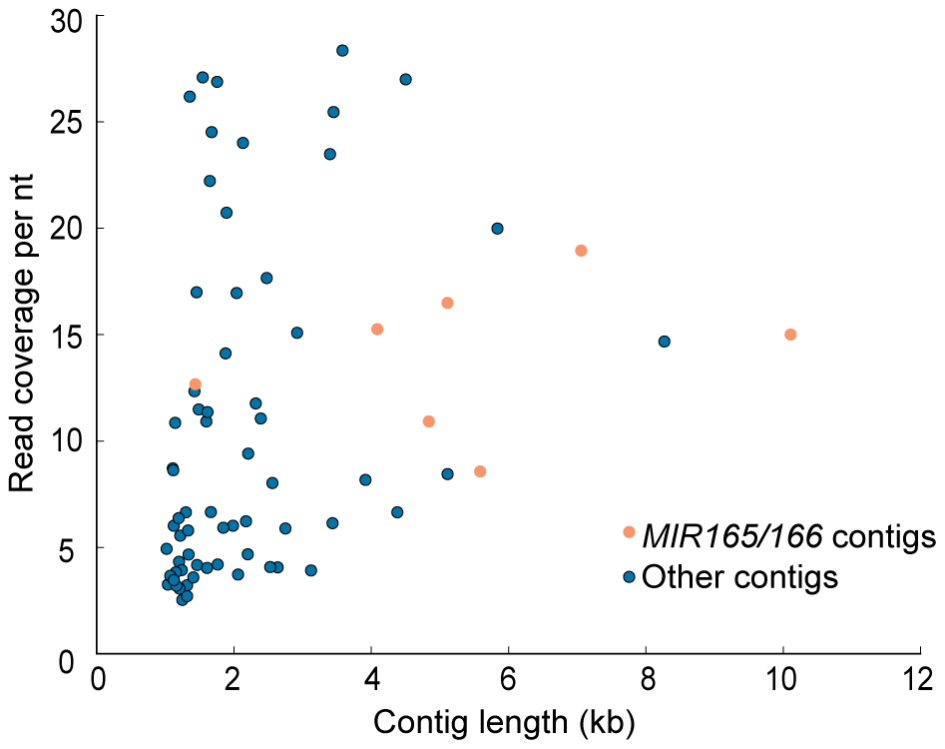
*de novo* assembly of enriched *MIR166* and *MIR165* loci. Coverage and length of contigs longer than one kb assembled by Velvet, using 1% of the paired-end data, are shown. Light brown dots: Contigs hosting a sequence targeted by the capture probe. Blue dots: Other contigs with no complementarity to the capture probe.

Reads were *de novo* assembled with the Velvet assembler [28] in order to test the feasibility to recover flanking sequences of the targeted loci in the absence of a reference sequence. Assembly proceeded using 1% of the total paired-end reads, which were randomly selected. All contigs greater than one kb in length and having sequence complementary to the capture probe (identified by BLASTn against the miR166 sequence) were indeed *MIR165/166* flanking regions (identified by BLASTn against the genome) (Fig. 6). Seven out of the eight *MIR165/166* loci were recovered in the assembled contigs, missing only *MIR166c/MIR166d*. This is likely due to the fact that *MIR166c/d* locus has the highest enrichment among all targeted loci, resulted from an additive effect of two target sites (Fig. S2, 4^th^ panel). We hypothesized that different coverage may affect the assembly result. Therefore we varied the number of reads fed into Velvet from 0.25% to 4% of the total reads (approximately 62k to 994k reads), resulting in a coverage per nt ranging from five to 80 at the assembled contigs, as was estimated by Velvet (Table 1). Indeed, the number of *MIR165/166* loci recovered in the assembled contigs changed with varying read coverage. Specifically, at the lower extreme of five reads per nt, the two *MIR165* loci, whose enrichment level were the lowest among all targets due to one mismatch to the capture probe, were missing in the assembled contigs. At the upper extreme of 80 reads per nt, none of the targeted loci were recovered, likely because at such a high coverage, the enriched loci were treated as repetitive regions by Velvet [29]. At the intermediate coverage levels, for example, ten reads per nt, all targets but *MIR165b* (lowest enrichment, Fig. S2, 2^nd^ panel) were recovered (Table S4). At 20 reads per nt, all but *MIR166c/d* (highest enrichment, Fig. S2, 4^th^ panel) were recovered (Table S4). Therefore, by combining the assembly result at both coverage levels, all targeted regions were assembled. Overall, Velvet is sensitive to the local read coverage near the targeted loci. However, by tuning the read coverage to the range of 10–20, we could assemble all the targeted loci.

**Table 1:** Velvet assembly result is sensitive to read coverage.

Percentage of total reads used in assembly	Number of reads	Coverage per nt	Number of *MIRNA* containing contigs (length>1000 bp)	*MIRNA*s recovered
4.0%	993,695	80	0	None
2.0%	497,844	40	2	*MIR166f* and *MIR165b*
1.0%*	248,489	20	7	All but *MIR166c/d*
0.5%*	124,550	10	8**	All but *MIR165b*
0.25%*	62,474	5	6	All but *MIR165a*, *MIR165b*

* At these levels of read coverage, three independent read sampling and assembly experiments were performed. All results were consistent.

** *MIR166c* and *MIR166d* were assembled into separate contigs, despite their ∼2 kb distance. See Table S4 for details.

Next, we evaluated the quality of the contigs matching the *MIR165/166* loci assembled from 1% and 0.5% of the total reads respectively. Contig sizes ranged from 1,639 bp to 11,652 bp, with a median of 5,499 bp (Table S4). Undetermined nucleotides in the contigs (originated from ‘N’s in the reads) accounted for about one third of the total differences between the contigs and the reference genome (Table S4). After removing all alignment positions with an N in the contigs, the percentage of mismatches to the reference genome was low, ranging from 0% to 1.56%, with a median of 0.17%. The percentage of gaps (single or multiple indels) was relatively high, ranging from 5.17% to 21.65%, with a median of 12.45%. However, most of the differences were caused by gaps larger than five nts (Table S4). The presence of large gaps in the assembly should not significantly affect the downstream analysis, if we apply this methodology to sample multiple plant genomes in order to study conserved CREs of *MIRNA*s. Because CREs are generally short [30], large gaps will only appear as missing information, rather than errors and noise that confound short motif identification.

### Trial enrichment experiments in *Zea mays* were unsuccessful

Given the success of the targeted enrichment method in *Arabidopsis*, we wanted to investigate its potential application to larger and more complex genomes. A targeted enrichment experiment, using the protocol optimized in *Arabidopsis*, was performed to enrich *MIR165/166* loci in *Zea mays* (maize), whose genome is highly repetitive and 17 times the size of *Arabidopsis* genome [31]. However, we failed to observe any significant enrichment in any of the targeted loci compared to control regions. Experimental conditions were explored to try to accommodate the difficulty of enrichment in a large, complex genome, including increasing hybridization temperature, increasing the amount of input gDNA, varying the probe-to-gDNA ratio, and applying a second round of enrichment. Unfortunately, none of the above attempts succeeded in enriching the targeted regions. An enrichment experiment performed with both *Arabidopsis* and maize in parallel ruled out technical errors as the reason for the failure in maize, as over ∼1,000 fold of enrichment was observed for an *Arabidopsis MIR166* locus, while enrichment was barely seen for two maize *MIR166* loci (Table S5). Therefore, we conclude that further optimization of the enrichment procedure will be required to extend this methodology into species with more complex and/or unknown genomes.

## Discussion

### A novel solution-based targeted genomic enrichment method successfully enriched large regions flanking targeted loci in *Arabidopsis*

We have shown the potential application of a novel solution-based targeted genomic enrichment method to enrich large flanking regions surrounding a known core sequence. Pilot experiments in *Arabidopsis* demonstrate the high specificity and sensitivity of this method to enrich sequences of interest. Successful *de novo* assembly of the sequencing reads into contigs covering the targeted loci indicated the feasibility to assemble the enriched regions in species with unknown genomes. This targeted genomic enrichment methodology is novel in several ways: First, it is the only existing enrichment method that relies solely on the knowledge of a short conserved core sequence. This method is especially suitable to study CREs of plant *MIRNAs*, because for deeply conserved loci, the ∼ 21 nt mature miRNA sequences are almost identical in multiple plant species [2], [3], while other regions of the primary transcripts are variable, and CREs are generally unknown. Since the capture probe can only be as long as the conserved sequence, i.e. 21 nt long in this project, a locked-nucleic acid (LNA)-modified probe is used to increase the thermostability of the probe-DNA-hybrid. Second, it aims to capture and enrich large genomic regions, evidently several kilobases long (Fig. 1B, Fig. 2E, Fig. 6). In order to achieve this goal, DNA extraction is performed with care to reduce physical shearing, and genomic DNA is not fragmented before capture. Third, unlike most other enrichment methods which require a reference genome for mapping and identification [22], this method aims to identify unknown sequences flanking a known core, therefore *de novo* assembly is required. This requirement poses challenges to the downstream data analysis. Finally, this method is designed to be applied to multiple species at the same time, in order to extract conservation information from multiple sequence alignments of the enriched regions. Other targeted enrichment methods are generally designed for a single genome [22], [26].

### Assembly does not require large numbers of reads

The *de novo* assembly results indicate that a small fraction of the reads generated from one lane of an Illumina GAIIx system is sufficient to assemble all targeted regions (Table 1), on the order of ∼10^5^ reads. This suggests that we could potentially bar-code a hundred samples in one sequencing run, or even more on higher-throughput instruments. Technical challenges need to be addressed for assembly of bar-coded samples, such as single nucleotide polymorphisms [32] which are expected to be abundant in flanking regions of the target sequences that are not under selection. One caveat in using the Velvet assembler is that its assembly result is sensitive to the read coverage (Table 1). We found that a coverage of 10–20 reads per nt at the targeted loci worked best.

Room for improvement exists in the assembly stage, including pre-assembly error correction and using transcriptome assemblers. Pre-assembly error correction by detection and removing low frequency k-mers have been shown to increase assembly quality [29], [33]. Removing low complexity reads in the pre-processing may reduce the error caused by Ns in the assembled contigs (Table S4). Transcriptome assemblers, which take account of the large variations in sequencing depth, may be able to resolve the issue of Velvet favoring regions of a narrow range of coverage [29]. However, using transcriptome assemblers to assemble genomic DNA may introduce unnecessary overheads, such as assembling regions of low coverage at the cost of large memory requirements, computational cost to consider strand information and splicing variants, which are not relevant for genomic DNA. Adapting transcriptome assemblers for assembly of long-range enrichment sequences is a goal for future study.

### Methodological improvements are necessary for application in unknown genomes

Our attempt to enrich targeted regions in maize failed, despite varying multiple experimental parameters. We think the failure might be due to the highly repetitive nature of the maize genome [31]. Indeed, an analysis of the 20 kb flanking regions of 12 maize *MIR166* loci showed an average 20mer frequency of 317 (Fig. S3A), while the average 20mer frequency of the 20 kb flanking regions was 6.6 for the nine *Arabidopsis MIR165/166* loci (Fig. S3B). It is possible that the targeted loci are indeed captured, but the repetitive sequences flanking the targeted loci hybridize with other repetitive sequences in the genome, and are captured and enriched together with the targeted loci. In the worst scenario, this could approach the capture of the entire genome, resulting in no enrichment at all. Alternatively, the failure of enrichment may be due to the fact that the maize genome is 20 times as large as *Arabidopsis* genome, and 11 distinct *MIR165/166* loci are present in *Z. mays* compared to eight in *Arabidopsis*, so the potential targeted sites are diluted to one fifteenth in *Z. mays*. However, increasing the input genomic DNA concentration and varying the probe-to-DNA ratio did not help. It is also possible that some intrinsic property of the maize genome hinders the hybridization between the probe and the target. In any case, more effort is needed before a general protocol can be developed in order to enrich sequences of interests in genomes with different size and complexity.

## Methods

### Targeted genomic enrichment experiment in *Arabidopsis*

Genomic DNA was extracted from wild-type *Arabidopsis thaliana* Col-0 leaves using Nucleon PhytoPure Genomic DNA Extraction Kits (GE Healthcare). 100 µg genomic DNA (100 µl @ 1 µg/1 µl), 300 µl Hybridization Buffer P5 (Invitrogen) and 1 pmole LNA-biotinylated capture probe (1 µl @ 1 µM) were placed in a 1.7 ml centrifuge tube and boiled for five minutes to denature the genomic DNA. The mix was incubated at 45°C for 30 minutes for hybridization. 20 µl of streptavidin beads from the RiboMinus Plant Kit (Invitrogen) were prepared per the manufacturer's protocol. After 30 minutes of hybridization, the hybridization mix was added to the beads and incubated at 45°C for 15 minutes with occasional (every two-three minutes) gentle mixing by inversion. Beads were captured with a magnetic stand and were washed three times each for two minutes with 500 µl 0.1X SSC incubated at 45°C. Captured DNA was eluted for one minute with 500 µl nanopure water at 90°C twice. DNA was mixed with 1/10 volume 3 M sodium acetate pH 5.2, 20 µg glycogen (1 µl @ 20 µg/μl) and three volumes of 95% ethanol, vortexed for 30 seconds, and then placed at −20°C overnight for ethanol precipitation. DNA was centrifuged at maximum speed at 4°C for 20 minutes to spin down pellets. Pellets were washed by 75% ethanol and centrifuged at maximum speed at 4°C for 5 minutes and air dried at 4°C. DNA was then resuspended in a minimal volume (4–8 µl) of nanopure water. DNA was linearly amplified with the Illustra GenomiPhi V2 DNA Amplification Kit (GE Healthcare) per the manufacturer's protocol, with the only modification that the incubation time was increased from 1.5 hours to 2 hours.

### Quantitative real-time PCR and data analysis

Real-time PCR was performed using a QuantiTect SYBR Green PCR kit (Qiagen) on a StepOne Real-Time PCR System (Applied Biosystems). Primers were designed to amplify regions with varying distances from one of the targeted loci, *MIR166a*. *Actin1* was used as a control, as *Actin1* is far from any of the targeted loci, therefore should not be enriched. All oligonucleotide sequences used in the study are listed in Table S6. For each primer set, two samples of captured and then linear-amplified DNA (captured DNA for short), as well as two samples of the diluted original genomic DNA were loaded. At the same time, serial dilution of the extracted genomic DNA was used. The method to calculate the normalized fold of enrichment is as follows. First, Ct values from the serial dilution experiments were used to calculate the linear relationship between Ct and log(Dilution) as Ct  =  A*log(Dilution) + b. Second, average Cts were taken for captured DNA and genomic DNA samples respectively, and the “pseudo dilution” values were calculated from the average Cts, A and b. Dividing the “pseudo dilution” value of the captured DNA by that of the genomic DNA resulted in the relative concentration of the specific targeted region in the captured DNA sample. Finally, the relative concentration of the targeted region was normalized by that of *Actin1* to calculate the normalized fold of enrichment.

### Paired-end sequencing and reference-based analysis

An *Arabidopsis* sample prepared with the targeted genomic enrichment methodology was fragmented to a mean size of ∼400 bp and paired-end sequenced on an Illumina GAIIx sequencer. Paired-end reads were mapped to the *Arabidopsis thaliana* reference genome (TAIR10) using Bowtie 0.12.7 [34] with parameters “-v2 -X500”. Non-uniquely mapped reads (12.93% of all mapped reads) were identified and one mapped location was randomly kept. Raw data have been deposited at NCBI SRA (accession SRX323012). Mapped reads were assigned to 1 kb-sized bins of the nuclear genome based on the midpoint of the mapping positions. Reads mapping to the chloroplast or mitochondria were discarded prior to analysis. Read coverage for each bin was defined as the number of reads assigned to that bin. Normalized coverage for each bin was defined as the read coverage of that bin divided by the nuclear genome average of the read coverage per bin. Enrichment is implied when the normalized coverage is above one. The threshold of normalized coverage by which a bin was considered “enriched” was determined by performance analysis, and a normalized coverage of ten was chosen by balancing sensitivity and specificity. Enriched bins were merged if within ten bins apart and extended ten bins to each side to define the surrounding regions of enriched bins. The Pearson correlation coefficient r was calculated to examine the linear dependence between |x| and log(y) where x is the distance to the most highly enriched bin in the 21 kb region centered on that bin and y is the normalized coverage. To assess the tolerance of mismatches between the probe and potential targets, the reference genome was scanned to identify sequences with different mismatch patterns. Then the normalized coverage of the bin where the sequence fell into was used to evaluate the effect mismatches had on enrichment, assuming the sequence was responsible for the enrichment.

### *de novo* assembly of the paired-end reads and assembly quality evaluation

Random samples from all the paired-end sequenced reads were generated by accepting each pair of reads at a given probability. For example, to generate 1% of the total reads, the acceptance probability is 0.01. Sampled reads were then *de novo* assembled with the Velvet assembler [28]. Parameters used for velveth were “31 -shortPaired -fastq” and parameters for velvetg were “exp_cov 20 ins_length 400 ins_length_sd 100”. However, we observed that changing “exp_cov” to “40” did not affect the assembly result. Assembled contigs were searched for complementary sequences to miR166 with BLASTn. All contigs harboring a miR166 matching sequence, together with all contigs long than 1000 bp, were BLASTed against the reference genome to identify the origin. Assembly quality of contigs from targeted loci were evaluated by first generating global alignment between the contig and corresponding sequence in the reference genome using the EMBOSS application needle [35] and then counting the number of mismatches, short gaps (defined as indels < =  five bp long) and long gaps (defined as indels > five bp long).

### Analysis of repetitiveness of *MIR165/166* flanking sequences in maize and *Arabidopsis*

The maize reference genome was retrieved from http://ftp.maizesequence.org/current/assembly/ and indexed with the suffixerator program in GenomeTools [36]. 20mer frequency across the genome was calculated using Tallymer [37] as an indicator of repetitiveness. 20mer frequency of the 20 kb flanking regions of 12 maize *MIR166* loci [38] was averaged and shown in Fig. S3A. The same analysis was performed for the 9 *Arabidopsis MIR165/166* loci and was shown in Fig. S3B.

## Supporting Information

Figure S1 **29 amplification time does not significantly affect the normalized fold change.** Quantitative real-time PCR (qPCR) shows that the normalized fold change relative to *Act1* after enrichment with different 29 amplification time at different distances flanking a targeted locus *MIR166a*.
(TIF)

Figure S2 **Normalized fold change in highly enriched regions and surrounding bins.** Each panel shows the normalized fold change at each 1 kb-sized bin centered on a highly enriched region. Genomic coordinates of the region and the Pearson correlation r are shown. Red line indicates the genome average of the normalized coverage, which equals one. Shaded panels are regions surrounding the eight *MIR165/166* loci (*MIR166c* and *MIR166d* are two bins apart, therefore are shown in the same panel).
(TIF)

Figure S3 **(A) Average 20mer frequency of the 20 kb flanking regions of 12 maize** ***MIR166*** **loci. (B) As in A for the nine** ***Arabidopsis MIR165/166*** **loci.**
(TIF)

Table S1 **Summary of mapped reads from long-range miR166 enrichment in** ***Arabidopsis thaliana*** **.**
(DOCX)

Table S2 **Performance analysis of varying threshold of normalized coverage to determine enriched regions.**
(DOCX)

Table S3 Pearson correlation coefficient r of |x|* and log(y)** of highly enriched regions is a good classifier of targeted and non-targeted loci.
(DOCX)

Table S4 **Quality of assembled** ***MIR165/166*** **contigs.**
(DOCX)

Table S5 **Quantitative real-time PCR results from an enrichment experiment in both** ***Arabidopsis*** **(Ath) and maize (Zma).** Ath *Act1*, Zma Actin and Zma *GAPDH* serve as controls. Primer sequences are listed in Table S6.
(DOCX)

Table S6 **Oligonucleotide sequences.**
(DOCX)
