# Supplementary figures and images for: Long-Range Genomic Enrichment, Sequencing, and Assembly to Determine Unknown Sequences Flanking a Known microRNA
Source: PLoS One. 2013 Dec 20;8(12):e83721. doi: 10.1371/journal.pone.0083721 (PMC3869802; doi:10.1371/journal.pone.0083721)

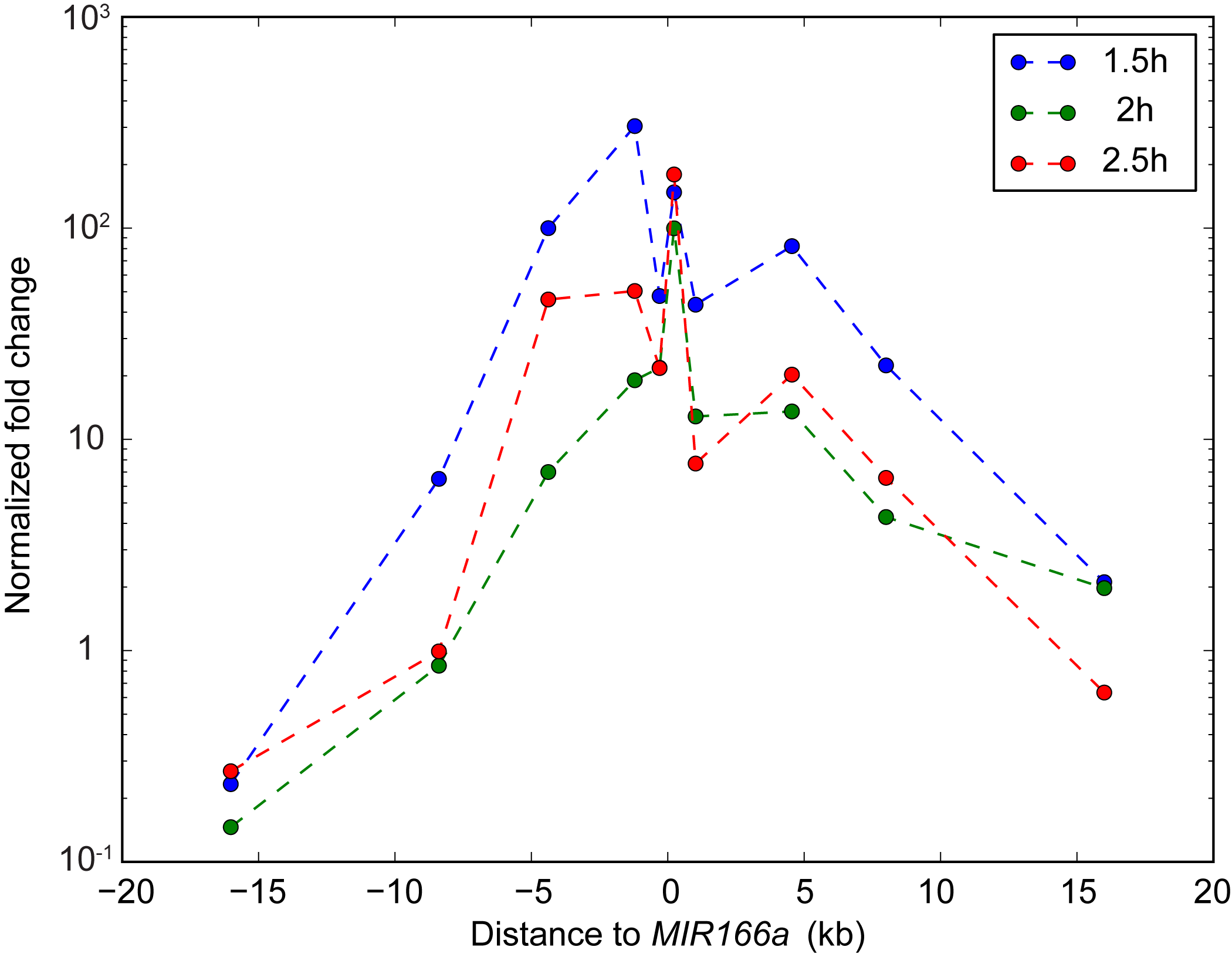

Supplement: Figure S1 — 29 amplification time does not significantly affect the normalized fold change. Quantitative real-time PCR (qPCR) shows that the normalized fold change relative to Act1 after enrichment with different 29 amplification time at different distances flanking a targeted locus MIR166a. (TIF) [file pone.0083721.s001.tif]

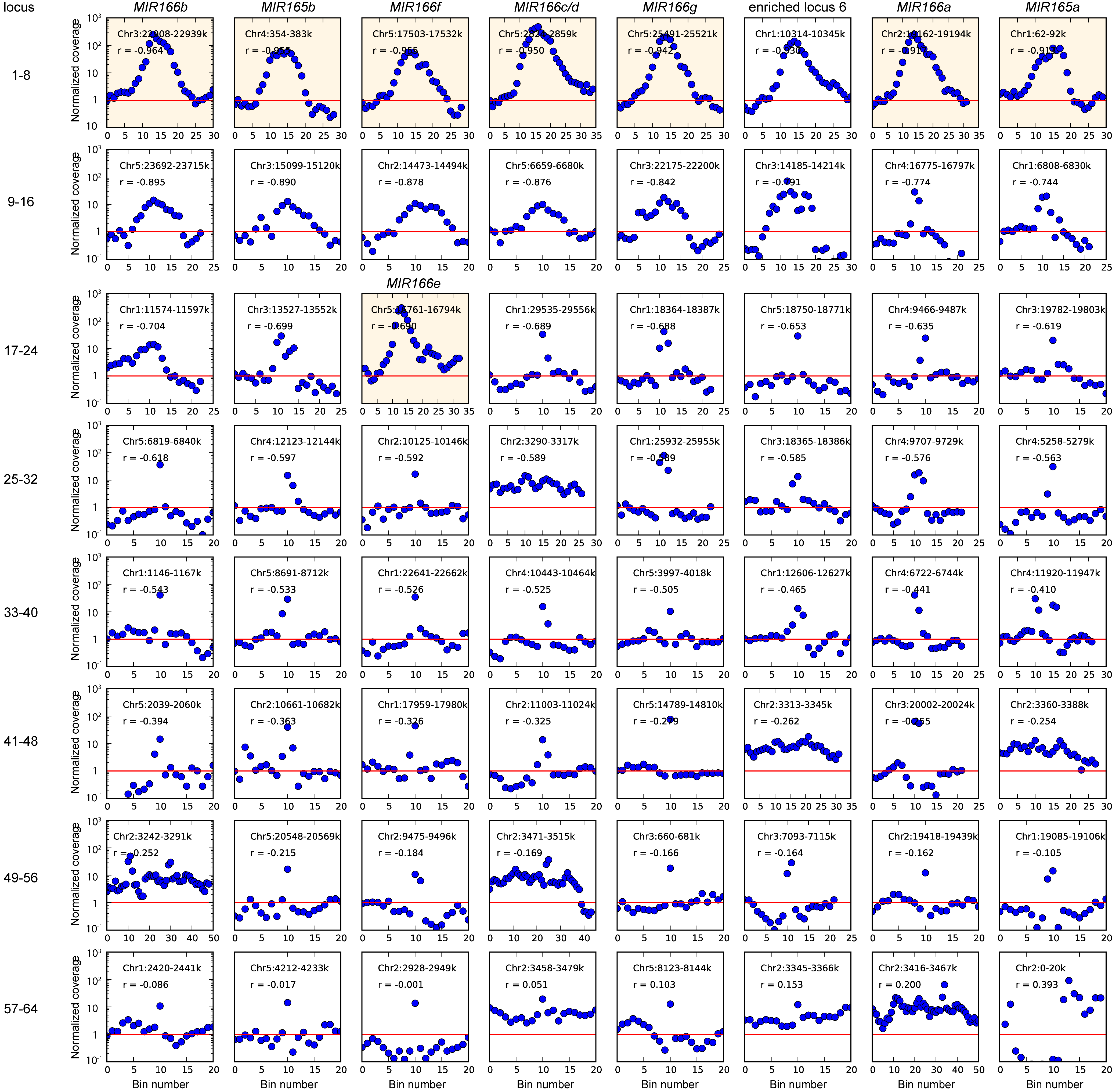

Supplement: Figure S2 — Normalized fold change in highly enriched regions and surrounding bins. Each panel shows the normalized fold change at each 1 kb-sized bin centered on a highly enriched region. Genomic coordinates of the region and the Pearson correlation r are shown. Red line indicates the genome average of the normalized coverage, which equals one. Shaded panels are regions surrounding the eight MIR165/166 loci (MIR166c and MIR166d are two bins apart, therefore are shown in the same panel). (TIF) [file pone.0083721.s002.tif]

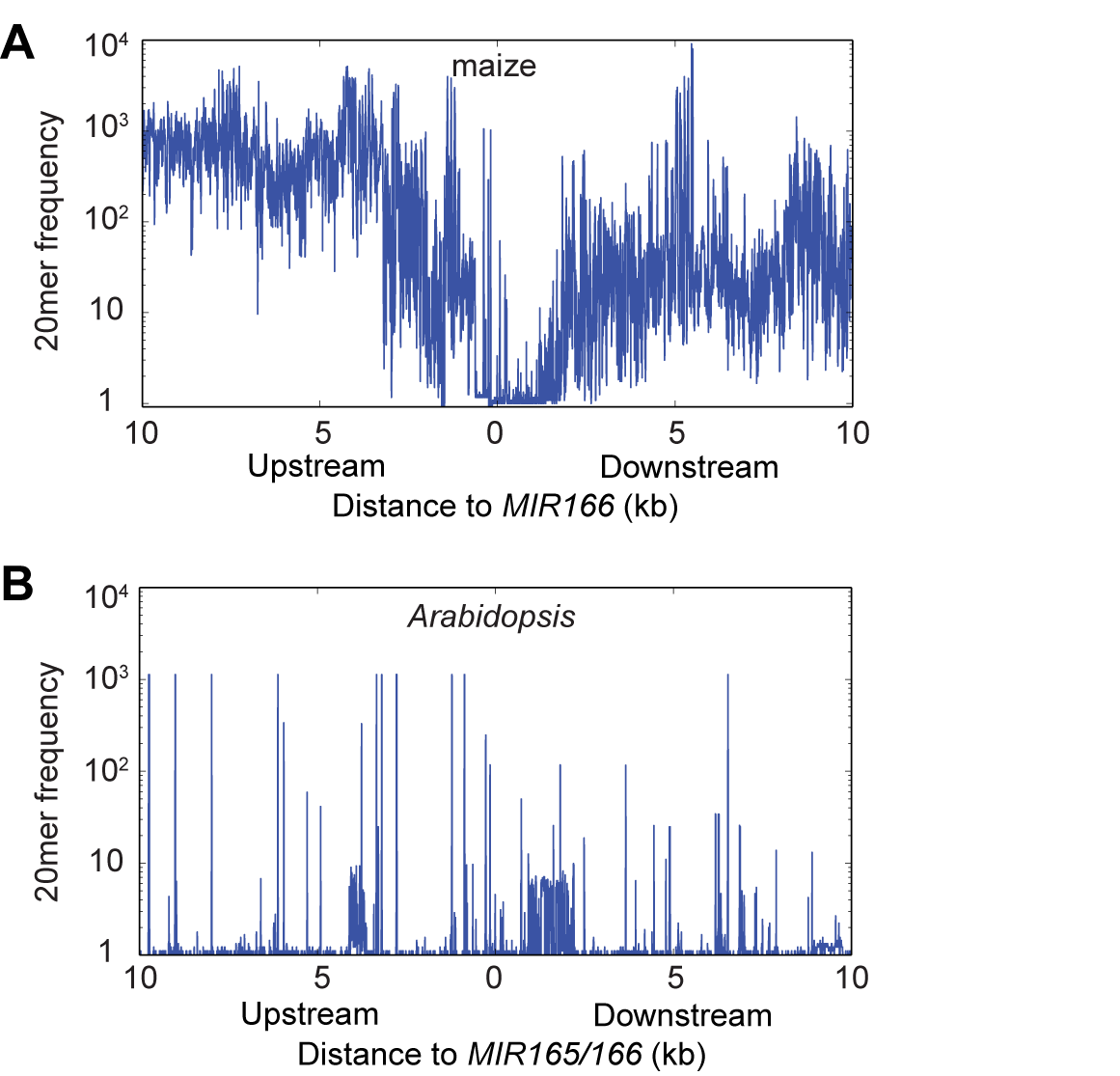

Supplement: Figure S3 — (A) Average 20mer frequency of the 20 kb flanking regions of 12 maize MIR166 loci. (B) As in A for the nine Arabidopsis MIR165/166 loci. (TIF) [file pone.0083721.s003.tif]
