# Supplementary material for: Long-Range Genomic Enrichment, Sequencing, and Assembly to Determine Unknown Sequences Flanking a Known microRNA
Source: PLoS One. 2013 Dec 20;8(12):e83721. doi: 10.1371/journal.pone.0083721 (PMC3869802; doi:10.1371/journal.pone.0083721)
Supplement: Table S1 — Summary of mapped reads from long-range miR166 enrichment in Arabidopsis thaliana . (DOCX) [file pone.0083721.s004.docx]

**Table S1.** Summary of mapped reads from long-range miR166 enrichment in *Arabidopsis thaliana*.

| Genome | Number of mapped reads | Percentage out of all mapped reads | Percentage out of all reads  (total: 24,859,558) |
| --- | --- | --- | --- |
| All | 17,548,544 | 100 | 70.59 |
| Nuclear genome | 11,426,625 | 65.11 | 45.97 |
| Plastid genome | 5,676,213 | 32.35 | 22.83 |
| Mitochondrial genome | 445,706 | 2.54 | 1.79 |
