# Supplementary material for: Long-Range Genomic Enrichment, Sequencing, and Assembly to Determine Unknown Sequences Flanking a Known microRNA
Source: PLoS One. 2013 Dec 20;8(12):e83721. doi: 10.1371/journal.pone.0083721 (PMC3869802; doi:10.1371/journal.pone.0083721)
Supplement: Table S2 — Performance analysis of varying threshold of normalized coverage to determine enriched regions. (DOCX) [file pone.0083721.s005.docx]

**Table S2.** Performance analysis of varying threshold of normalized coverage to determine enriched regions.

| Within 5kb from target site as true positives | | | | | | | |
| --- | --- | --- | --- | --- | --- | --- | --- |
| Threshold (normalized coverage) | True positive (TP) | False negative (FN) | False positive (FP) | True negative (TN) | Sensitivity (TP/(TP+FN)) | Specificity (TN/(TN+FP)) | |
| 100 | 22 | 57 | 7 | 119,063 | 0.2785 | 0.9999 | |
| 50 | 36 | 43 | 17 | 119,053 | 0.4557 | 0.9999 | |
| 20 | 56 | 23 | 61 | 119,009 | 0.7089 | 0.9995 | |
| 10* | 68 | 11 | 153 | 118,917 | 0.8608 | 0.9987 | |
| 5 | 72 | 7 | 636 | 118,434 | 0.9114 | 0.9947 | |
| 2 | 76 | 3 | 8,104 | 110,966 | 0.9620 | 0.9319 | |
| 1 | 78 | 1 | 37,556 | 81,514 | 0.9873 | 0.6846 | |
| 0.5 | 79 | 0 | 83,131 | 35,939 | 1.0000 | 0.3018 | |
| 0.2 | 79 | 0 | 112,713 | 6,357 | 1.0000 | 0.0534 | |
| Within 9kb from target site as true positives | | | | | | | |
| Threshold (normalized coverage) | True positive (TP) | False negative (FN) | False positive (FP) | True negative (TN) | Sensitivity (TP/(TP+FN)) | Specificity (TN/(TN+FP)) |  |
| 100 | 22 | 113 | 7 | 119,007 | 0.1630 | 0.9999 |  |
| 50 | 36 | 99 | 17 | 118,997 | 0.2667 | 0.9999 |  |
| 20 | 59 | 76 | 58 | 118,956 | 0.4370 | 0.9995 |  |
| 10* | 76 | 59 | 145 | 118,869 | 0.5630 | 0.9988 |  |
| 5 | 89 | 46 | 619 | 118,395 | 0.6593 | 0.9948 |  |
| 2 | 107 | 28 | 8,073 | 110,941 | 0.7926 | 0.9322 |  |
| 1 | 124 | 11 | 37,510 | 81,504 | 0.9185 | 0.6848 |  |
| 1 | 132 | 3 | 83,078 | 35,936 | 0.9778 | 0.3019 |  |
| 0 | 135 | 0 | 112,657 | 6,357 | 1.0000 | 0.0534 |  |

* Threshold chosen for further analysis.
