# Supplementary material for: Long-Range Genomic Enrichment, Sequencing, and Assembly to Determine Unknown Sequences Flanking a Known microRNA
Source: PLoS One. 2013 Dec 20;8(12):e83721. doi: 10.1371/journal.pone.0083721 (PMC3869802; doi:10.1371/journal.pone.0083721)
Supplement: Table S3 — Pearson correlation coefficient r of |x|* and log(y)** of highly enriched regions is a good classifier of targeted and non-targeted loci. (DOCX) [file pone.0083721.s006.docx]

**Table S3.** Pearson correlation coefficient r of |x|* and log(y)** of highly enriched regions is a good classifier of targeted and non-targeted loci.

| Locus number*** | Genome coordinates | Pearson correlation r | *MIR165/166* loci |
| --- | --- | --- | --- |
| 1 | 3:22908-22939k | -0.964 | Y |
| 2 | 4:354-383k | -0.955 | Y |
| 3 | 5:17503-17532k | -0.955 | Y |
| 4 | 5:2824-2859k | -0.950 | Y |
| 5 | 5:25491-25521k | -0.942 | Y |
| 6 | 1:10314-10345k | -0.930 | N |
| 7 | 2:19162-19194k | -0.917 | Y |
| 8 | 1:62-92k | -0.913 | Y |
| 9 | 5:23692-23715k | -0.895 | N |
| 10 | 3:15099-15120k | -0.890 | N |
| 11 | 2:14473-14494k | -0.878 | N |
| 12 | 5:6659-6680k | -0.876 | N |
| 13 | 3:22175-22200k | -0.842 | N |
| 14 | 3:14185-14214k | -0.791 | N |
| 15 | 4:16775-16797k | -0.774 | N |
| 16 | 1:6808-6830k | -0.744 | N |
| 17 | 1:11574-11597k | -0.704 | N |
| 18 | 3:13527-13552k | -0.699 | N |
| 19 | 5:16761-16794k | -0.690 | Y |
| 20 | 1:29535-29556k | -0.689 | N |
| 21 | 1:18364-18387k | -0.688 | N |
| 22 | 5:18750-18771k | -0.653 | N |
| 23 | 4:9466-9487k | -0.635 | N |
| 24 | 3:19782-19803k | -0.619 | N |
| 25 | 5:6819-6840k | -0.618 | N |
| 26 | 4:12123-12144k | -0.597 | N |
| 27 | 2:10125-10146k | -0.592 | N |
| 28 | 2:3290-3317k | -0.589 | N |
| 29 | 1:25932-25955k | -0.589 | N |
| 30 | 3:18365-18386k | -0.585 | N |
| 31 | 4:9707-9729k | -0.576 | N |
| 32 | 4:5258-5279k | -0.563 | N |
| 33 | 1:1146-1167k | -0.543 | N |
| 34 | 5:8691-8712k | -0.533 | N |
| 35 | 1:22641-22662k | -0.526 | N |
| 36 | 4:10443-10464k | -0.525 | N |
| 37 | 5:3997-4018k | -0.505 | N |
| 38 | 1:12606-12627k | -0.465 | N |
| 39 | 4:6722-6744k | -0.441 | N |
| 40 | 4:11920-11947k | -0.410 | N |
| 41 | 5:2039-2060k | -0.394 | N |
| 42 | 2:10661-10682k | -0.363 | N |
| 43 | 1:17959-17980k | -0.326 | N |
| 44 | 2:11003-11024k | -0.325 | N |
| 45 | 5:14789-14810k | -0.279 | N |
| 46 | 2:3313-3345k | -0.262 | N |
| 47 | 3:20002-20024k | -0.255 | N |
| 48 | 2:3360-3388k | -0.254 | N |
| 49 | 2:3242-3291k | -0.252 | N |
| 50 | 5:20548-20569k | -0.215 | N |
| 51 | 2:9475-9496k | -0.184 | N |
| 52 | 2:3471-3515k | -0.169 | N |
| 53 | 3:660-681k | -0.166 | N |
| 54 | 3:7093-7115k | -0.164 | N |
| 55 | 2:19418-19439k | -0.162 | N |
| 56 | 1:19085-19106k | -0.105 | N |
| 57 | 1:2420-2441k | -0.086 | N |
| 58 | 5:4212-4233k | -0.017 | N |
| 59 | 2:2928-2949k | -0.001 | N |
| 60 | 2:3458-3479k | 0.051 | N |
| 61 | 5:8123-8144k | 0.103 | N |
| 62 | 2:3345-3366k | 0.153 | N |
| 63 | 2:3416-3467k | 0.200 | N |
| 64 | 2:0-20k | 0.393 | N |

* x is the distance to the most highly enriched bin in the 21 kb region centered on that bin.

** y is the normalized coverage.

*** See Fig. S2 for plots of all loci.
