# Supplementary material for: Long-Range Genomic Enrichment, Sequencing, and Assembly to Determine Unknown Sequences Flanking a Known microRNA
Source: PLoS One. 2013 Dec 20;8(12):e83721. doi: 10.1371/journal.pone.0083721 (PMC3869802; doi:10.1371/journal.pone.0083721)
Supplement: Table S4 — Quality of assembled MIR165/166 contigs. (DOCX) [file pone.0083721.s007.docx]

**Table S4.** Quality of assembled *MIR165/166* contigs.

| Matching locus | Contig length | # mis-matches* | % mis-matches* | # nt in  gaps* | % nt in  gaps  * | % nt in gaps > 5 nt** | # mis-matches due to N | % mis-matches due to N | # nt in gaps  due to N | % nt in gaps due to N | Assembled from % of total reads |
| --- | --- | --- | --- | --- | --- | --- | --- | --- | --- | --- | --- |
| *MIR166a* | 11,652 | 22 | 0 | 1,636 | 14 | 98 | 27 | 0 | 1,248 | 11 | 1 |
| *MIR166b* | 5,394 | 1 | 0 | 284 | 5 | 100 | 0 | 0 | 84 | 2 | 1 |
| *MIR166e* | 4,758 | 74 | 2 | 762 | 16 | 88 | 2 | 0 | 378 | 8 | 1 |
| *MIR166f* | 6,355 | 0 | 0 | 770 | 12 | 100 | 0 | 0 | 569 | 9 | 1 |
| *MIR166g* | 7,442 | 7 | 0 | 385 | 5 | 98 | 0 | 0 | 167 | 2 | 1 |
| *MIR165a* | 5,456 | 17 | 0 | 621 | 11 | 98 | 10 | 0 | 413 | 8 | 1 |
| *MIR165b* | 1,639 | 6 | 0 | 204 | 12 | 97 | 0 | 0 | 0 | 0 | 1 |
| *MIR166a* | 5,499 | 57 | 1 | 733 | 13 | 93 | 5 | 0 | 311 | 6 | 0.5 |
| *MIR166b* | 7,398 | 16 | 0 | 430 | 6 | 97 | 11 | 0 | 170 | 2 | 0.5 |
| *MIR166c* | 3,007 | 5 | 0 | 592 | 20 | 99 | 6 | 0 | 342 | 11 | 0.5 |
| *MIR166d* | 6,861 | 7 | 0 | 570 | 8 | 97 | 2 | 0 | 351 | 5 | 0.5 |
| *MIR166e* | 7,225 | 12 | 0 | 1,328 | 18 | 99 | 1 | 0 | 739 | 10 | 0.5 |
| *MIR166f* | 1,889 | 1 | 0 | 312 | 17 | 100 | 0 | 0 | 112 | 6 | 0.5 |
| *MIR166g* | 6,096 | 7 | 0 | 1,320 | 22 | 100 | 0 | 0 | 604 | 10 | 0.5 |
| *MIR165a* | 4,925 | 6 | 0 | 491 | 10 | 98 | 2 | 0 | 279 | 6 | 0.5 |

* These calculations exclude alignment positions where the nucleotide of the contig is N.

** Percentage of nucleotides in gaps > 5nt out of all nucleotides in gaps.
