# Supplementary material for: Long-Range Genomic Enrichment, Sequencing, and Assembly to Determine Unknown Sequences Flanking a Known microRNA
Source: PLoS One. 2013 Dec 20;8(12):e83721. doi: 10.1371/journal.pone.0083721 (PMC3869802; doi:10.1371/journal.pone.0083721)
Supplement: Table S5 — Quantitative real-time PCR results from an enrichment experiment in both Arabidopsis (Ath) and maize (Zma). Ath Act1, Zma Actin and Zma GAPDH serve as controls. Primer sequences are listed in Table S6. (DOCX) [file pone.0083721.s008.docx]

**Table S5.** Quantitative real-time PCR results from an enrichment experiment in both *Arabidopsis* (Ath) and maize (Zma). Ath *Act1*, Zma Actin and Zma *GAPDH* serve as controls. Primer sequences are listed in Table S6.

| Distance to nearest targeted locus | PCR-amplified region in Ath | Median fold of enrichment relative to Ath *Act1* | PCR-amplified region in Zma | Median fold of enrichment relative to Zma *GAPDH* |
| --- | --- | --- | --- | --- |
| ~1k | *MIR166a* Close | 7466.3 | *MIR166c* Close | 18.9 |
| ~4k | *MIR166a* Far | 1976.9 | *MIR166c* Far | 11.1 |
| ~1k | / | / | *MIR166m* Close | 1.7 |
| > 200k | *Act1* | 1 | *Actin* | 2.3 |
| > 200k | / | / | *GAPDH* | 1 |
