# Supplementary material for: Long-Range Genomic Enrichment, Sequencing, and Assembly to Determine Unknown Sequences Flanking a Known microRNA
Source: PLoS One. 2013 Dec 20;8(12):e83721. doi: 10.1371/journal.pone.0083721 (PMC3869802; doi:10.1371/journal.pone.0083721)
Supplement: Table S6 — Oligonucleotide sequences. (DOCX) [file pone.0083721.s009.docx]

**Table S6.** Oligonucleotide sequences

| **Name** | | **Oligonucleotide sequence** | |
| --- | --- | --- | --- |
| LNA-modified capture probe complementary to miR166 | | GGGGAATGAAGCCTGGTCCGA/3Bio/ | |
| ***Arabidopsis* locus** | **Distance to *MIR166a* (kb) or nearest targeted locus** | **Forward primer sequence** | **Reverse primer sequence** |
| -0.06kb_*MIR166a* | -0.06 | GAGGACTCTGGCTCGCTCTAT | CCGAAGACGCTAAAACCCTAAT |
| -0.30kb_*MIR166a* | -0.3 | CTTTTCTTTGGCTCTCTCCACTAC | GGCTAAGATTAAGGGAGAAGCAG |
| -0.46kb_*MIR166a* | -0.46 | AGTCCAGTGACATGTCTCAAAAAG | CCACACGCTCTCTCTAACAATACA |
| -1.21kb_*MIR166a* | -1.21 | GTATCTCTTTCACCGGATGTTCTT | CGACTAGCTTTTACCCAAACAA |
| -4.38kb_*MIR166a* (*MIR166a* Far) | -4.38 | CCTTGAATACATAAGAAACGGTAGC | CAAATTTCCAACCCCATAAGTC |
| -8.39kb_*MIR166a* | -8.39 | ACAAGTTTACCTTCACCACACGTA | CTTTTTGTAAGGAAGGGGTCAAC |
| -16.02kb_*MIR166a* | -16.02 | TGTCCAACTTTGAGTATGATGTGG | CCTGTCCTCTTTAGTTCGTCAAAT |
| -32.83kb_*MIR166a* | -32.83 | GTTTTAGTCTTTCTTTGACGCTGAC | GTTCAAATAGTTCGTGTTGTGGAC |
| -64.03kb_*MIR166a* | -64.03 | CCTTAACGAAATCCAAAAGCTG | GATGCAGATGCTATCGTTCTAGTT |
| -128.04kb_*MIR166a* | -128.04 | GATTAAAGTTGTCGGATTTCTCAGC | ATCATTTTATCTCCCCCGTTACAC |
| 0.23kb_*MIR166a* (*MIR166a* Close) | 0.23 | TAAATAGGTCTTTGGGGACCGATA | CAGATTCCAAGAGGAGAAAGAAAG |
| 1.02kb_*MIR166a* | 1.02 | CGGCGTTGAGAAACACTTTACTA | AGACGCTCTCTATTTCTTGACG |
| 2.07kb_*MIR166a* | 2.07 | ATTCGCAAATGGGCATCATA | ATTCTAAAGCCGGTTCTAATAGGG |
| 4.55kb_*MIR166a* | 4.55 | AGCTCTCTCGAACTCACATCTCTT | GAAGAAACCAAGAAAGACCAAGAAG |
| 8.00kb_*MIR166a* | 8 | CCTCAAGGCTATATGACCTGTGTAA | TCCTTATAGCTGCCTACTAGTGAAA |
| 16.00kb_*MIR166a* | 16 | GACAAATACGAGAACATCCACAAC | GGATCTTGACGCGTGTGAG |
| 32.19kb_*MIR166a* | 32.19 | GCATATAGACGATGGCTGTTACTG | GGAAAAGTGTACAAAGAGGCTAGG |
| 63.98kb_*MIR166a* | 63.98 | TACAGGCAAACACTGGTTAGTACC | GTCTCTTATTTCGTTAACCCGTGA |
| 127.99kb_*MIR166a* | 127.99 | ACTGTCTGTAGCTGAATTGCGTAT | TTACGACACATGTACTCACCTCAA |
| *Act1* | > 200 | ATGTTCCCTGGAATCGCCGACAGA | AGGCCAAGATGGACCCTCCAATCC |
| **Maize locus** | **Distance to nearest targeted locus (kb)** | **Forward primer sequence** | **Reverse primer sequence** |
| *MIR166c* Close | ~ 1 | TCAATGATCGTACACAAACAAGTG | CCAAAGGGTGAGCATATTTTTATC |
| *MIR166c* Far | ~ 4 | AGCATAATGGTTTGGGTTAGTTGT | CAGTATCATAAATTGGAACGAACG |
| *MIR166m* Close | ~ 1 | AAAGCTTGCCCAGAACAGAA | TGAAGACTGCGGAATGTCTG |
| *Actin* | > 200 | CAATGGCACTGGAATGGT | ATCTTCAGGCGAAACACG |
| *GAPDH* | > 200 | CCACATGAACAGCACCAAGT | GTCACTCAATGCTCAACCAA |
